# Regulation of Postnatal Trabecular Bone Formation by the Osteoblast Endothelin A Receptor

**DOI:** 10.1002/jbmr.450

**Published:** 2011-06-22

**Authors:** Gregory A Clines, Khalid S Mohammad, Jessica M Grunda, Katrina L Clines, Maria Niewolna, C Ryan McKenna, Christopher R McKibbin, Masashi Yanagisawa, Larry J Suva, John M Chirgwin, Theresa A Guise

**Affiliations:** 1 Department of Medicine, Division of Endocrinology, Diabetes and Metabolism, University of Alabama at BirminghamBirmingham, AL, USA; 2 Veterans Affairs Medical CenterBirmingham, AL, USA; 3 Department of Medicine, Division of Endocrinology and Metabolism, University of VirginiaCharlottesville, VA, USA; 4 Department of Molecular Genetics and Howard Hughes Medical Institute, University of Texas Southwestern Medical CenterDallas, TX, USA; 5 Department of Orthopaedic Surgery, Center for Orthopaedic Research, University of Arkansas for Medical SciencesLittle Rock, AR, USA

**Keywords:** ENDOTHELIN A RECEPTOR, ENDOTHELIN-1, OSTEOBLAST, BONE, SEX STEROIDS

## Abstract

Endothelin-1 (ET-1) is a potent vasoconstrictor that also stimulates cells in the osteoblast lineage by binding to the endothelin A receptor (ETAR). ET-1 ligand is widely secreted, particularly by the vasculature. However, the contributions of ETAR signaling to adult bone homeostasis have not been defined. ETAR was inactivated in osteoblasts by crossing ETAR-floxed and osteocalcin-*Cre* mice. Histomorphometric analyses were performed on 4-, 8-, and 12-week-old osteoblast-targeted ETAR knockout (KO) and wild-type (WT) male and female mice. Tibial trabecular bone volume was significantly lower from 12 weeks in KO versus WT mice in both males and females. Bone-formation rate, osteoblast density, and in vitro osteoblast differentiation were reduced by targeted inactivation of ETAR. A separate longitudinal analysis was performed between 8 and 64 weeks to examine the effect of aging and castration on bone metabolism in ETAR KO mice. Hypogonadism did not change the rate of bone accrual in WT or KO females. However, eugonadal KO males had a significantly larger increase in tibial and femoral bone acquisition than WT mice. Male mice castrated at 8 weeks of age showed the reverse: KO mice had reduced rates of tibial and femoral BMD acquisition compared with WT mice. In vitro, ET-1 increased osteoblast proliferation, survival, and differentiation. Dihydrotestosterone also increased osteoblast differentiation using a mechanism distinct from the actions of ET-1. These results demonstrate that endothelin signaling in osteoblasts is an important regulator of postnatal trabecular bone remodeling and a modulator of androgen effects on bone. © 2011 American Society for Bone and Mineral Research

## Introduction

Endothelin-1 (ET-1) is a multifunctional 21-amino-acid peptide vasoconstrictor that has effects on cancer and bone.^1^, ^2^ ET-1 binds to two similar seven-transmembrane-domain G protein–coupled receptors, the endothelin A receptor (ETAR) and the endothelin B receptor (ETBR). ETAR appears to transmit most of the biologic signals of ET-1, with ETBR frequently serving as a clearance receptor.

In 1995, Nelson and colleagues reported that secreted ET-1 was responsible for the formation of disorganized new bone characteristic of skeletal metastases owing to prostate cancers,^3^ providing the first major evidence of the physiologic importance of the endothelin axis in bone. Bone metastases are a cooperative interaction between tumor and host, where tumor cells flourish in the hospitable bone microenvironment and bone undergoes pathologic remodeling under the direction of secreted tumor factors such as ET-1. Evidence supportive of this model includes increased circulating ET-1 in men with advanced prostate cancer with bone metastases^3^ and ET-1 secretion by breast cancer cell lines that produced osteoblastic lesions in an animal model of bone metastasis.^4^ The ETAR-selective antagonist atrasentan blocked the formation of osteoblastic lesions in breast and prostate cancer animal models of bone metastasis.^4^, ^5^ In separate human clinical trials, atrasentan and zibotentan, another ETAR-selective antagonist, reduced the progression of osteoblastic bone metastases in men with advanced prostate cancer.^6^, ^7^ The effects of ETAR blockade on bone unaffected by cancer are unknown.

ET-1 acts via autocrine/paracrine stimulation of Wnt signaling in bone. Canonical Wnt signaling is a critical pathway for osteoblast differentiation and bone development.^8^–^11^ Dickkopf homologue 1 (DKK1), a secreted inhibitor of canonical Wnt signaling, was decreased in a gene array search for targets of ET-1 in calvarial bone.^12^ Recombinant DKK1 blocked ET-1-mediated osteoblast proliferation and new bone formation, whereas ET-1 stimulated nuclear translocation of β-catenin in osteoblasts.^12^ The data collectively support a mechanism where the anabolic action of ET-1 occurs through Wnt activation consequent to decreased inhibitory DKK1. Other ET-1 regulators and targets, such as endothelin-converting enzyme 1,^13^ calcineurin/NFAT activation,^14^ and secretion of vasculae endothelial factor F (VEGF)^15^ and bone morphogenetic protein 7 (BMP-7),^16^ may have additional effects on osteoblasts to increase bone formation.

The importance of ET-1 in the pathology of osteoblastic metastases and the identification of osteoblast signaling pathways activated by ET-1 in vitro suggests that a local ET-1 axis is critical for normal bone development and remodeling. Further, since ETAR antagonists may be administered to patients with cancer in the future, it is important to determine the effects on bone unaffected by cancer in order to optimize skeletal health. To test this hypothesis, ETAR was genetically inactivated in osteoblasts using Cre-Lox technology. Global deletion of ET-1 or ETAR in mice is lethal,^17^, ^18^ requiring tissue-specific inactivation. The bone phenotypes of young and aged mice were studied in both sexes. We hypothesized that sex steroids modulate endothelin signaling and tested the effects of gonadectomy. Our results indicated that osteoblast ETAR is important in postnatal bone formation in both males and females and that androgens modulate ETAR action and bone acquisition in older male mice. In vitro studies revealed that ET-1 indirectly increased osteoblast differentiation by downregulating DKK1 secretion, which was restricted to mature osteoblasts. ET-1, in cooperation with androgens, also directly increased differentiation of osteoprogenitors independently of DKK1 and canonical Wnt signaling. These results have important implications for future therapeutic applications of ETAR antagonists for advanced prostate cancer in men who are also treated by androgen-deprivation therapy.

## Materials and Methods

### Animals

Animal experimentation followed approved protocols and the guidelines of the Institutional Animal Care and Use Committees of the University of Virginia and the University of Alabama at Birmingham. Generation of transgenic osteocalcin-*Cre* (Oc*Cre*)^19^ and knock-in ETAR *loxP* mice (ETAR*loxP*)^20^ mice has been described. Mice heterozygous for (Oc*Cre*^+/−^) were crossed with homozygous ETAR *loxP* mice (ETAR*loxP*^+/+^). Progeny were genotyped for Oc*Cre* and ETAR*loxP*. One-half the progeny carried the Oc*Cre*^+/−^/ETAR*loxP*^+/−^ genotype. Males carrying both *Cre* and *loxP* may undergo promiscuous *loxP* recombination owing to *Cre* misexpression within the germ line, resulting in non-tissue-specific ETAR inactivation. Oc*Cre*^+/−^/ETAR*loxP*^+/−^ females therefore were backcrossed to ETAR*loxP*^+/+^ males. Progeny from this cross contain one of four expected genotypes (Oc*Cre*^+/−^/ETAR*loxP*^+/+^, Oc*Cre*^+/−^/ETAR*loxP*^+/−^, Oc*Cre*^−/−^/ETAR*loxP*^+/+^, or Oc*Cre*^−/−^/ETAR*loxP*^+/−^). Two genotypes were selected for study: Oc*Cre*^+/−^/ETAR*loxP*^+/+^ and Oc*Cre*^−/−^/ETAR*loxP*^+/+^ littermate controls. Young mice were euthanized at 4, 8, or 12 weeks of age. Aged mice were euthanized at 64 weeks of age. Bones were harvested for analysis. The size of each group ranged from 5 to 10 mice. Eight-week-old male and female mice underwent sham surgery or castration using standard procedures.

### Genotyping

A piece of the tail (5 mm) was cut from 3-week-old mice. Total genomic DNA was prepared using the DirectPCR Lysis Reagent (Viagen Biotech, Los Angeles, CA, USA) according to the manufacturer's recommendations. The Oc*Cre* transgene was identified by PCR using the following primers: *Ost1*: CAA ATA GCC CTG GCA GAT; and *RBG1*: TGA TAC AAG GGA CAT CTT CC. A 300-bp PCR product confirmed the presence of the transgene. The ETAR *loxP* knock-in cassette was identified using the following primers: *ETAExo*: CCT CAG GAA GGA AGT AGC AAG ATT A; and *RAF102*: ACA CAA CCA TGG TGT CGA. The wild-type ETAR allele produced a 610-bp product and the *loxP* cassette produced a 650-bp product.

### Histomorphometry

Thoracolumbar vertebrae and forelimb and hind limb long bones were removed from mice following euthanasia, fixed in 10% buffered formalin, decalcified in 10% EDTA, paraffin embedded, cut at 3.5 µm, and stained. Bone histomorphometric analyses were performed with the MetaMorph imaging and software system (Universal Imaging Corp., Sunnyvale, CA, USA) Trabecular bone indices were determined: trabecular bone volume (BV/TV), trabecular number (Tb.N), trabecular thickness (Tb.Th), and trabecular separation (Tb.Sp). Osteoblasts and osteoclasts were counted using a ×20 objective (number of cells/0.24-mm^2^ area) in the proximal tibia and distal femur below the primary spongiosa. Osteoblasts were identified as large cells aligned as a palisade along the bone surface. Osteoclasts were identified by tartrate-resistant acid phosphatase (TRACP) staining. Standard histomorphometric nomenclature was used and followed American Society for Bone and Mineral Research recommendations.^21^

### Immunohistochemistry

Tibias from 4-week-old ETAR WT and KO mice were sectioned and deparafinized. Sections were incubated with 0.3% hydrogen peroxide for 30 minutes, washed in PBS, treated with Protein Blocking Agent (Immunotech, Marseille, France) for 2 hours at room temperature, and then incubated for 30 minutes with a rabbit anti-ETAR antibody (Alomone Labs, Jerusalem, Israel) or a mouse anti-active β-catenin antibody (clone 8E7; Millipore, St Charles, MO, USA) at a concentration of 1:10 or 1:500, respectively. Slides were washed with PBS and incubated with a biotin-conjugated secondary antibody (Vector Laboratories, Burlingame, CA, USA) for 30 minutes. Vectastain ABC Reagent (Vector Laboratories) was used for detection. Slides were counterstained with hematoxylin. Cytoplasmic and nuclear β-catenin staining intensities were analyzed using MetaMorph (Universal Imaging Corp.). Controls using secondary antibody without primary antibody showed no staining (data not shown).

### Micro–computed tomography (µCT)

Trabecular bone volume and skeletal microarchitecture in the left tibial metaphysis of each mouse were measured ex vivo by µCT (µCT40, Scanco Medical, Bassersdorf, Switzerland) using the manufacturer's software. All µCT analyses were consistent with current guidelines for the assessment of bone microstructure in rodents using µCT.^22^ Cross-sectional images were obtained with a voxel size of 16 µm in each dimension. Semiautomated contouring was used to select a region of interest (ROI) comprising the secondary spongiosa and extending 3.2 mm distal to the primary spongiosa but excluding cortex and subcortical bone, composed of 150 adjacent 16-µm slices. For calculation of the 3D volume and architecture of the secondary spongiosa, the volume of each slice was stacked before application of an optimized Gaussian noise filter and gray-scale threshold, manually determined to be 245.^23^ Trabecular bone volume (BV/TV) and architectural parameters (trabecular thickness [Tb.Th], number [Tb.N], and separation [Tb.Sp] and connectivity density [Conn.D]) were calculated directly from the reconstructed trabecular structures.^24^

### Dynamic histomorphometry

Before euthanasia, mice underwent calcein-tetracycline-calcein labeling in order to visualize bone formation. Calcein (0.02 mg/g of body weight) was administered intraperitoneally 10 and 3 days before euthanasia, and tetracycline (0.03 mg/g of body weight) was administered intraperitoneally 7 days before euthanasia. Lumbar spines were fixed in 10% buffered formalin for 24 hours and placed in 70% ETOH. The bones were embedded in methyl methacrylate and sectioned at 7 µm. Using a fluorescence microscope, bone-formation and mineral apposition rates were calculated.^25^

### Bone marrow culture

Tibias, femurs, and humeruses were harvested from ETAR*loxP*^+/+^ and ETAR*loxP*^−/−^ mice. Bone marrow was flushed and cells counted. For fibroblast and osteoblast colony-forming unit (CFU-F and CFU-OB) assays, 1 × 10^6^ cells were plated in 35-mm dishes in α-MEM/15% fetal bovine serum (FBS)/100 IU/mL of penicillin and 100 µg/mL of streptomycin in quadruplicate. Two days after plating, 50 µg/mL of ascorbic acid and 10 mM β-glycerophosphate were added to the medium. In addition, either adenovirus *Cre* or a control adenovirus (Vector Biolabs, Philadelphia, PA, USA) at a multiplicity of infection (MOI) of 5 was added. The medium was changed every 2 to 3 days. Cells were either stained for alkaline phosphatase (SigmaFast BCIP/NBT, Sigma-Aldrich, St. Louis, MO, USA) at 12 days (CFU-F) or alizarin red (CFU-OB) at 30 days after plating.

For osteoclast assays, 2 × 10^6^ bone marrow cells containing both osteoblast and osteoclast precursors were plated in 0.95-cm^2^ wells using α-MEM without ribonucleosides, 10% FBS, 100 IU/mL of penicillin, 100 µg/mL of streptomycin, and 10 nM 1,25-dihydroxyvitamin D_3_ [1.25(OH)_2_D_3_].^26^, ^27^ Two days after plating, either adenovirus *Cre* or a control adenovirus (Vector Biolabs, Philadelphia, PA, USA) at an MOI of 5 was added to the medium. Cultures then were stained for TRACP (Sigma, St Louis, MO, USA) 8 days after plating.

### DXA analysis

BMD of mice was determined by dual-energy X-ray absorptiometry (DXA) using a GE Lunar PIXImus II (GE Healthcare, Madison, WI, USA) bone densitometer at 8, 12, 16, 20, 24, 28, 36, 44, 52, and 64 weeks of age. The densitometer was calibrated with a plastic-embedded murine phantom before use. Mice were anesthetized and placed on an adhesive tray in a prone position with limbs spread, and total body measurement was performed, excluding calvaria, mandible, and teeth. A region of interest was defined at the distal femur and proximal tibia just beneath the growth plate (12 × 12 pixels) and at the lumbar spine (20 × 50 pixels). Values were expressed as percent change in BMD over baseline in milligrams per square centimeter.

### In vitro analyses

Primary osteoblasts were grown as described previously.^12^ MC3T3-E1 (subclone 4) cells were obtained from American Type Culture Collection (ATCC) (Manassas, VA, USA) and grown in α-MEM containing 10% fetal calf serum, 1 mM sodium pyruvate, and 100 IU/mL of penicillin and 100 µg/mL of streptomycin. Calvarial organ cultures were performed as described previously^12^ and analyzed using BioQuant analysis software (BioQuant Image Analysis Corporation, Nashville, TN, USA). To induce osteoblast differentiation, 50 µg/mL of ascorbic acid and 10 mM β-glycerophosphate were added to the medium.

Standard treatments included 10 µM atrasentan (Abbott Laboratories, Abbott Park, IL, USA), 100 nM ET-1 (American Peptide, Sunnyvale, CA, USA), 50 ng/mL of recombinant mouse DKK1 (R&D Systems, Minneapolis, MN, USA), 10 nM 5α-dihydrotestosterone (DHT; Sigma-Aldrich, St Louis, MO, USA), and 10 nM 17β-estradiol (E_2_; Sigma-Aldrich). In experiments using DHT and E_2_, the cell culture medium was phenol red–free, and charcoal-stripped fetal calf serum was used (Invitrogen, Carlsbad, CA, USA). Alkaline phosphatase staining used a SigmaFast BCIP/NBT kit according to manufacturer's directions (Sigma-Aldrich). Staining intensity was determined using a UVP BioSpectrum Imaging System (Upland, CA, USA) to capture images and BioQuant to analyze intensity using the integrated optical density application. DKK1 and ET-1 protein concentrations were determined using ELISA (R&D Systems).

For cell cycle analysis, osteoblasts were washed and fixed in 70% ETOH. Fixed and permeabilized cells were treated with 40 µg/mL of propidium iodide and 200 µg/mL of RNase at 37 °C for 30 minutes. Apoptosis was determined using a FITC annexin V apoptosis kit according to the manufacturer's directions (BD Biosciences, San Jose, CA, USA). Cell cycle and apoptosis analyses were performed using a BD LSR II Analytical Flow Cytometer (BD Biosciences).

MC3T3 cells were transfected with TOPFlash or FOPFlash Wnt reported vectors (Millipore) plus *Renilla* luciferase as normalization standard. Twenty-four hours after transfection, cells were treated with factors, and dual luciferase assays were performed after 48 hours using a BioTek Synergy 2 microplate reader (Winooski, VT, USA).

### Statistical analysis

Statistical analyses were performed using Prism 4.00 software (Graphpad Software, La Jolla, CA, USA). Comparisons of two groups were performed using an unpaired two-tailed *t* test. Comparisons of three or more groups were performed using one-way ANOVA; post hoc analyses were performed using Tukey multiple-comparison testing. Longitudinal DXA data were analyzed by two-way ANOVA. Significant differences are indicated (*p < 0.05; **p < 0.01; ***p < 0.001).

## Results

### Osteoblast ETAR-targeted deletion strategy

ETAR was inactivated in the osteoblast using the Cre-Lox strategy of targeted gene deletion because of the embryonic lethality of global ETAR deletion^18^ and the multiple potential sources of ET-1 within the bone microenvironment. Osteocalcin-*Cre* (Oc*Cre*) transgenic mice contain the *Cre* recombinase expressed under the transcriptional control of the osteocalcin promoter,^19^ which is specifically activated late during osteoblast differentiation. Knock-in mice containing *loxP* sites flanking the last three exons of ETAR were generated previously.^20^ In the presence of *Cre*, exons 6 to 8 are deleted through *loxP* recombination, resulting in functional inactivation of the receptor. The Oc*Cre*^+/−^ and ETAR*loxP*^+/+^ mice were crossed in a two-step breeding strategy that produced mice with an osteoblast-targeted deletion of ETAR (Oc*Cre*^+/−^/ETAR*loxP*^+/+^, KO) and littermate controls without ETAR deletion (Oc*Cre*^−/−^/ETAR*loxP*^+/+^, WT).

Bones of WT and KO mice were examined in both young and aged mice. Twelve groups of young mice (WT versus KO, male versus female at 4, 8, and 12 weeks of age) and eight groups of aged (WT versus KO, male versus female, eugonadal versus hypogonadal) were studied. KO mice were of similar weight as WT mice and lacked visible anatomic abnormalities (data not shown).

### ETAR expression in bone

**Fig. 1:**
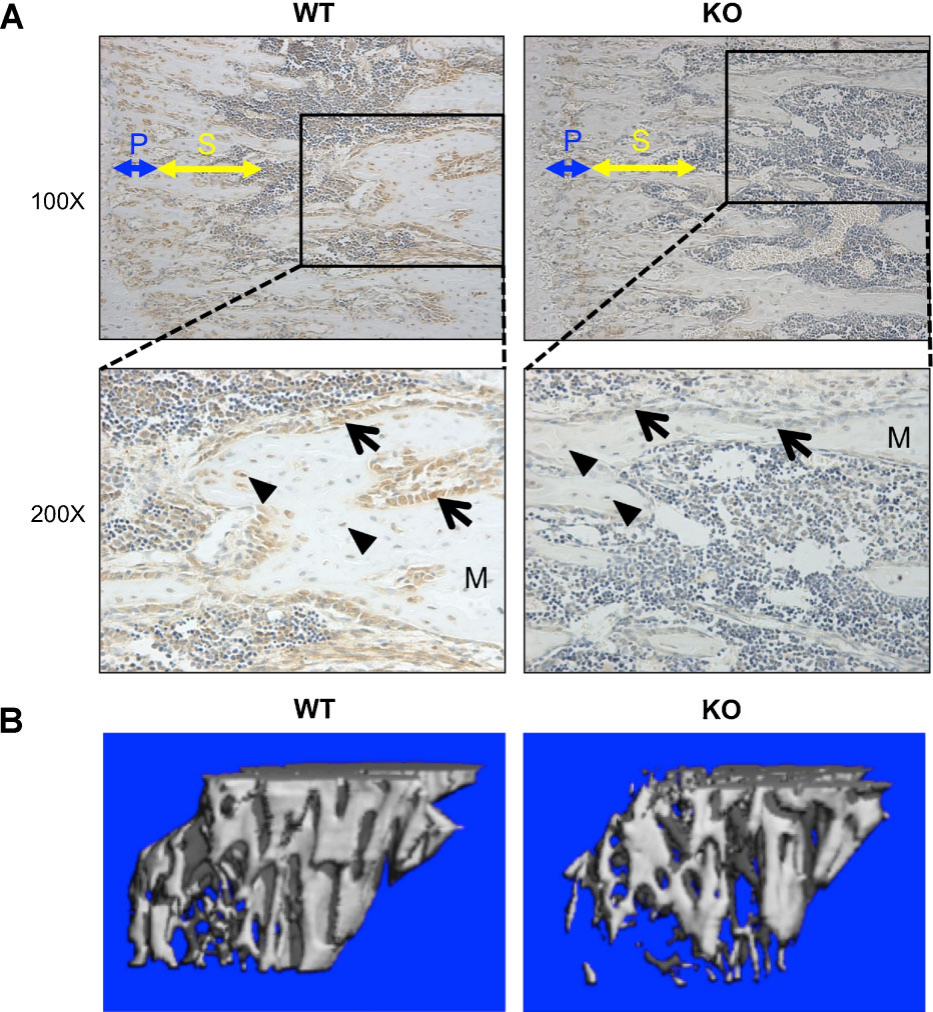
Reduced ETAR expression and trabecular bone volume in KO mouse tibias. (*A*) Tibias of WT and KO mice were analyzed for ETAR protein expression by immunohistochemistry. In ×100 magnification images, primary (*blue*) and secondary (*yellow*) spongiosae are indicated. Inset boxes correspond to ×200 magnification images. Arrows indicate osteoblasts, and triangles indicate osteocytes. M = bone matrix. (*B*) µCT images showing reconstructions of proximal tibia trabecular bone demonstrating lower trabecular bone volume in KO mice.

Biologic and functional evidence supported the expression of ETAR in murine calvarial and primary osteoblast cultures.^4^ However, its expression pattern in long bones was unknown. By immunohistochemistry (IHC), ETAR was detected in osteoblasts of the primary and secondary spongiosa and in trabecular osteoblasts in WT mice (Fig. 1*A*). Scant staining was seen in osteocytes. KO mice demonstrated significantly less ETAR staining in osteoblasts, confirming Cre-mediated inactivation of ETAR.

### Structural bone phenotype of young male mice

**Fig. 2:**
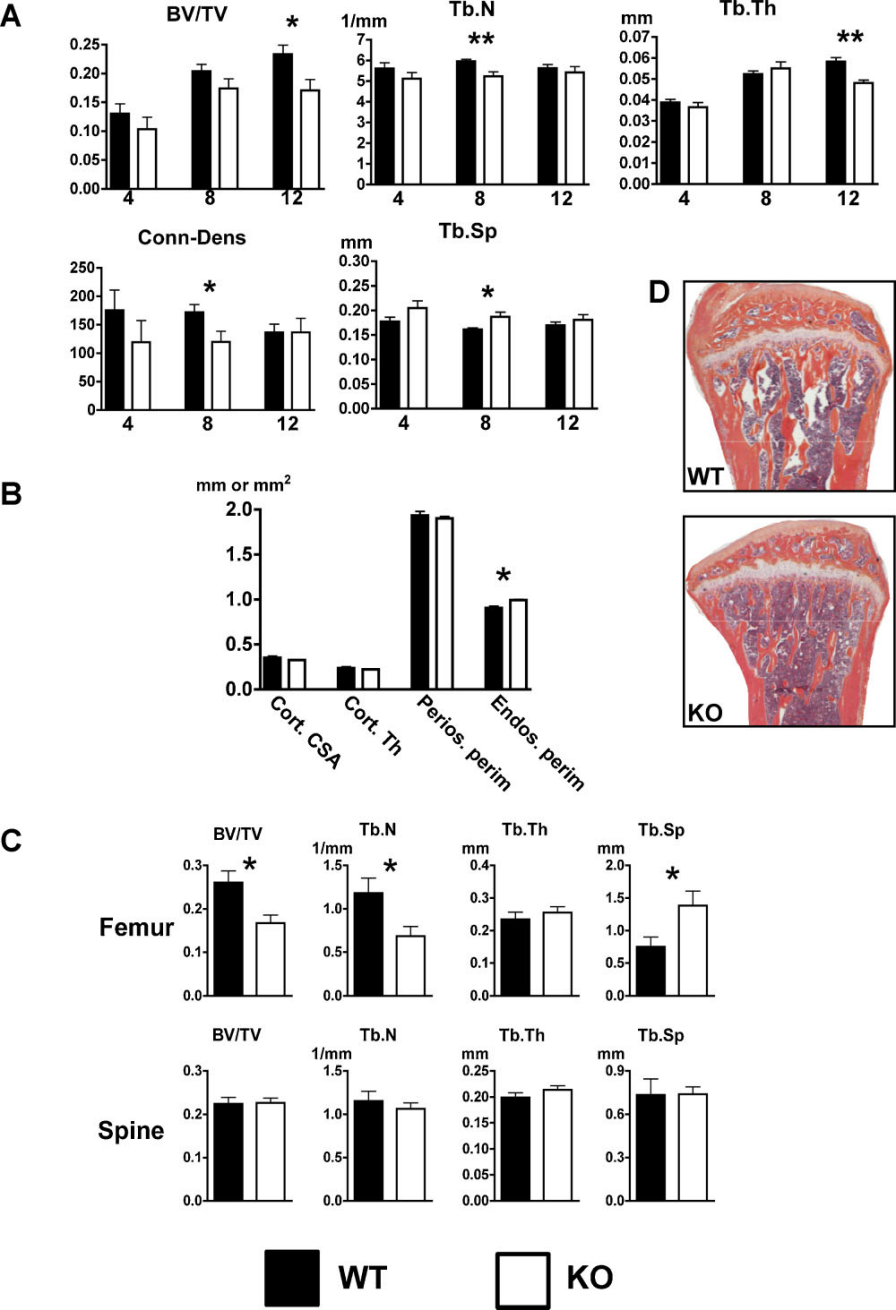
Lower tibia and femur trabecular bone volume in 12-week-old male KO mice. (*A*) The tibias of 4-, 8-, and 12-week-old male WT and KO mice were analyzed by µCT. Trabecular bone volume (BV/TV), trabecular number (Tb.N), trabecular thickness (Tb.Th), connectivity density (Conn.D), and trabecular separation (Tb.Sp) were determined. (*B*) Tibia cortical bone analysis of 12-week-old male mice revealed that KO mice had a slightly increased endosteal perimeter. Cortical cross-sectional area (Cort.CSA), cortical thickness (Cort.Th), periosteal perimeter (Perios.perim), and endosteal perimeter (Endos.perim) were determined. (*C*) Static bone histomorphometric analysis demonstrated reduced femoral, but not spine, trabecular bone volume in 12-week-old KO male mice. (*D*) Representative tibias from WT and KO mice stained with H&E/orange G (*p ≤ 0.05; **p ≤ 0.01).

Proximal tibias of 4-, 8-, and 12-week-old males were analyzed by µCT. Trabecular bone volume (TBV, BV/TV) was significantly reduced in KO mice at 12 weeks of age compared with controls (0.17 versus 0.23, *p* = 0.025; Figs. 1*B* and 2*A*); TBV was not significantly different at 4 and 8 weeks of age among the groups. Trabecular number (Tb.N) and connectivity density (Conn.D) were lower, and trabecular separation was higher in 8-week-old KO versus WT males, but was not enough to impact trabecular bone volume. Trabecular thickness (Tb.Th) was significantly lower at 12 weeks. Cortical bone parameters were examined in tibias of 12-week-old males. No significant differences in cortical cross-sectional area, thickness, or periosteal perimeter were measured. However, KO males had a larger endosteal perimeter (0.99 versus 0.90 mm, *p* = 0.013; Fig. 2*B*), suggesting lower endocortical osteoblast activity.

Because tibial TBV was significantly different at 12 weeks but not at earlier time points, we selected the 12-week time point to examine changes in femoral and thoracic spine trabecular bone by histomorphometry. Similar to the proximal tibial parameters, the distal femoral TBV was significantly lower in KO versus WT male mice (0.17 versus 0.26, *p* = 0.018; Fig. 2*C*). Tb.N and Tb.Sp also were significantly different. No significant differences in thoracic spine trabecular bone parameters were found (Fig. 2*C*).

### Structural bone phenotype of young female mice

**Fig. 3:**
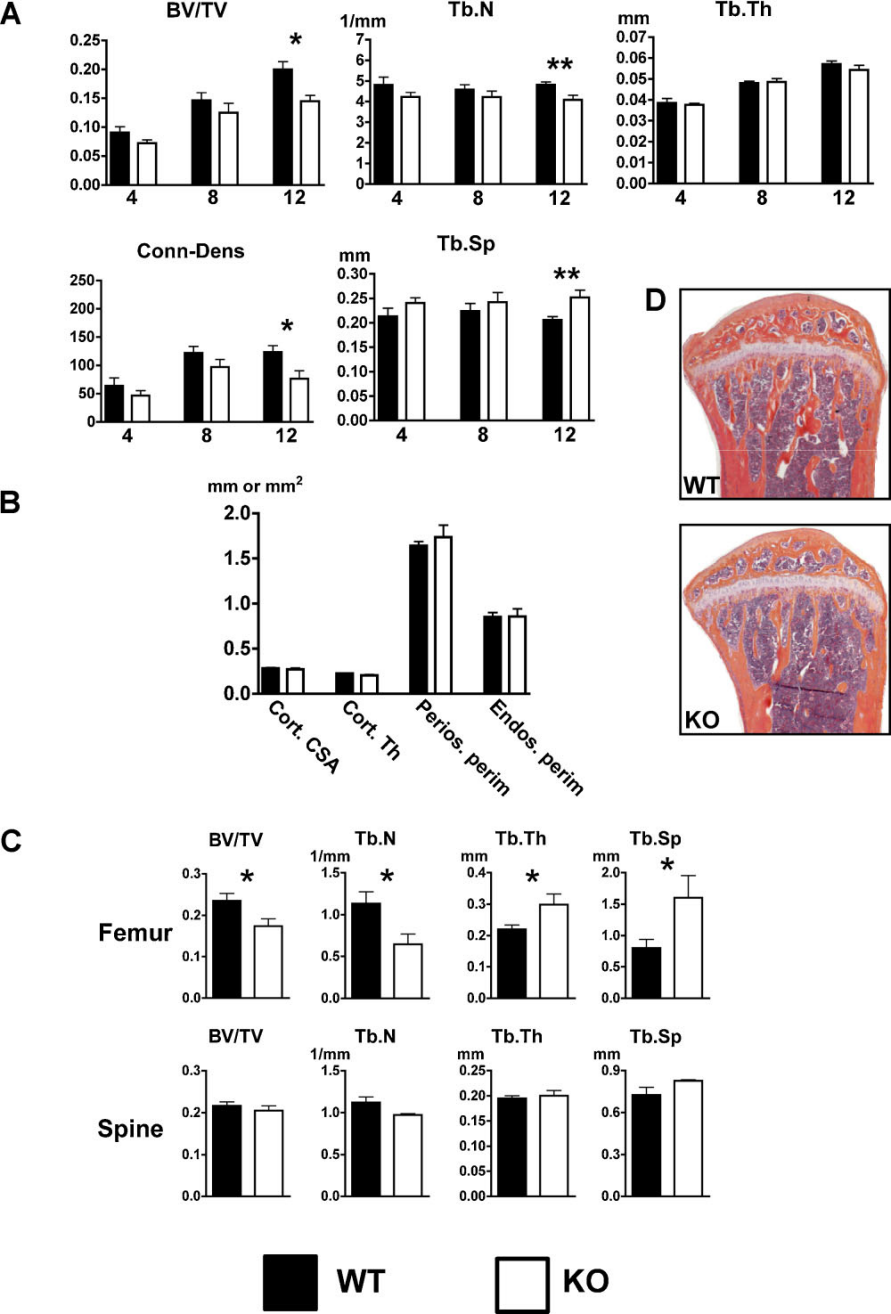
Reduced tibia and femur trabecular bone volume in 12-week-old female KO mice. (*A*) The tibias of 4-, 8-, and 12-week-old female WT and KO mice were analyzed by µCT. Trabecular bone volume (BV/TV), trabecular number (Tb.N), trabecular thickness (Tb.Th), connectivity density (Conn.D), and trabecular separation (Tb.Sp) were determined. (*B*) Tibia cortical bone analysis of 12-week-old female mice revealed that osteoblast-targeted ETAR KO did not alter cortical parameters by µCT. Cortical cross-sectional area (Cort.CSA), cortical thickness (Cort.Th), periosteal perimeter (Perios.perim), and endosteal perimeter (Endos.perim) were determined. (*C*) Static bone histomorphometric analysis demonstrated reduced femoral, but not spine, trabecular bone volume in 12-week-old KO female mice. (*D*) Representative tibias from WT and KO mice stained with H&E/orange G (*p ≤ 0.05; **p ≤ 0.01).

A parallel strategy was used to analyze the bone phenotype in female mice. Similar to male mice, TBV was significantly lower at 12 weeks (0.15 versus 0.20, *p* = 0.014) but not at 4 or 8 weeks (Fig. 3*A*). Tb.N and Conn.D also were lower, and the separation between trabeculae was higher only at 12 weeks. No differences were seen in cortical geometry in 12-week-old females (Fig. 3*B*).

Similar to male mice, femoral trabecular bone volume was lower in KO mice (0.18 versus 0.24, *p* = 0.036; Fig. 3*C*). Both Tb.N and Tb.Sp were consistent with lower volume of trabecular bone. Tb.Th was higher in female KO mice, but the greater decrease in Tb.N produced an overall reduction in trabecular bone, suggesting that signaling from the ETAR in osteoblasts may affect trabecular number more than trabecular thickness in females. As in male mice, no significant differences in thoracic spine trabecular bone parameters were seen in females (Fig. 3*C*).

### In vivo and ex vivo cellular and functional assays

**Fig. 4:**
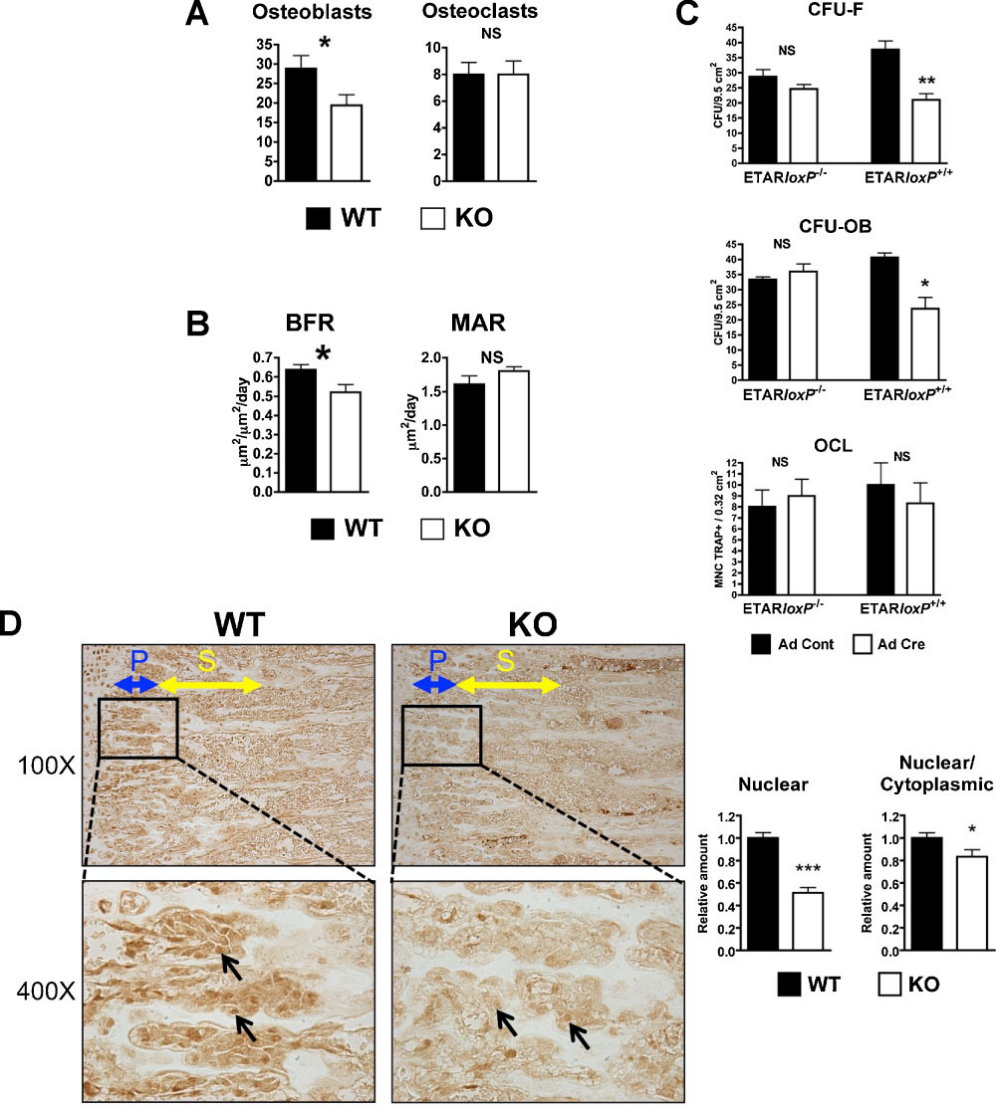
Lower osteoblast number, bone-formation rate, and osteoblast differentiation in KO mice. (*A*) Cellular density in 4-week-old mice tibias was determined by counting the number of osteoblasts and osteoclasts (TRACP staining) per ×200 field (0.24-mm^2^ area). (*B*) Dynamic histomorphometry was performed in 12-week-old mice measuring bone-formation rate (BFR) and mineral apposition rate (MAR). (*C*) Bone marrow cells were isolated from ETAR*loxP*^−/−^ or ETAR*loxP*^+/+^ mice and treated with adenovirus expressing *Cre* (Ad *Cre*) or control adenovirus (Ad Cont). Bone marrow culture analysis was performed for colony-forming unit fibroblast (CFU-F), colony-forming unit osteoblast (CFU-OB), and osteoclast formation (OCL). (*D*) Reduced osteoblast nuclear β-catenin and β-catenin nuclear/cytoplasmic ratio in KO mice. Tibias of 4-week-old WT and KO males were stained for the active fraction of β-catenin. Images were obtained immediately below the growth plate, and primary (*blue*) and secondary (*yellow*) spongiosae are indicated in ×100 magnification images. Inset boxes indicate area of ×400 magnification. Arrows indicate osteoblasts. The ×400 images were digitized. Multiple osteoblasts within the primary spongiosa were analyzed for nuclear and cytoplasmic β-catenin and staining intensity quantified (*p ≤ 0.05; **p ≤ 0.01; ***p ≤ 0.001).

No significant differences were found by histomorphometry in either the number of osteoblasts or osteoclasts on the trabecular bone surface of 12-week-old KO versus WT mice (data not shown). Cell numbers then were examined in mice at 4 weeks, when cellular density in bone is higher. When male and female animals were analyzed as separate groups, a trend toward a lower number of osteoblasts per unit volume of bone was seen in KO versus WT mice (data not shown). However, when male and female data were combined, ETAR WT mice had significantly more osteoblasts than KO mice (28.8 versus 19.4 cells/0.24 mm^2^, *p* = 0.044; Fig. 4*A*). No significant difference was found in number of osteoclasts (as TRACP^+^ multinucleated cells) per unit of bone surface (Fig. 4*A*).

Bone-formation rate (BFR) and mineral apposition rate (MAR) were calculated in 12-week-old male mice by the separation of calcein and tetracycline labels. BFR, but not MAR, was significantly decreased in KO mice (0.52 versus 0.64 µm^2^/µm^2^/d, *p* = 0.043; Fig. 4*B*). The data indicate that the rate of bone mineralization per osteoblast-formation unit was unchanged, whereas the total number of osteoblasts that form bone was lower with ETAR-targeted deletion.

The effects of ETAR deletion on osteoblast progenitor commitment and proliferation and on osteoclast formation were examined in vitro. Bone marrow was flushed from ETAR*loxP*^−/−^ or ETAR*loxP*^+/+^ mice, cultured, and transduced with adenovirus expressing Cre recombinase or control adenovirus. Cells were cultured in mineralization medium and assayed for alkaline phosphatase activity after 7 days or for mineralization after 28 days. Cre expression did not change the number of colonies in ETAR*loxP*^−/−^ bone marrow cultures. However, Cre expression in ETAR*loxP*^+/+^ osteoblasts significantly reduced the number of osteoblast progenitors (CFU-F; 21.0 versus 37.7 CFU/well, *p* = 0.01) and mature osteoblasts (CFU-OB; 23.7 versus 40.7 CFU/well, *p* = 0.014) compared with ETAR*loxP*^−/−^ osteoblasts (Fig. 4*C*). ETAR deletion did not affect osteoclastogenesis (Fig. 4*C*). The data are similar to the results in vivo, indicating that ETAR deletion reduces osteoblast differentiation and number but not osteoclastic bone resorption, leading to a net reduction in TBV.

The mechanism for reduced osteoblast differentiation and bone formation was examined. ET-1 increased osteoblastic bone formation via decreased DKK1, resulting in activation of the canonical Wnt signaling pathway.^12^ The degree of Wnt signaling activation was measured by IHC using an antibody specific for the nonphosphorylated or active form of β-catenin. Nuclear staining of osteoblasts in the primary spongiosa was 49% less in KO than in WT mice (Fig. 4*D*). To control for variation in staining, the relative nuclear-to-cytoplasmic ratio of each image also was measured and found to be 17% lower in KO than in WT mice. The results are consistent with the observations in vitro and in vivo and indicate that deletion of the ETAR reduces canonical Wnt signaling in active osteoblasts.

### Bone phenotype in aging mice

**Fig. 5:**
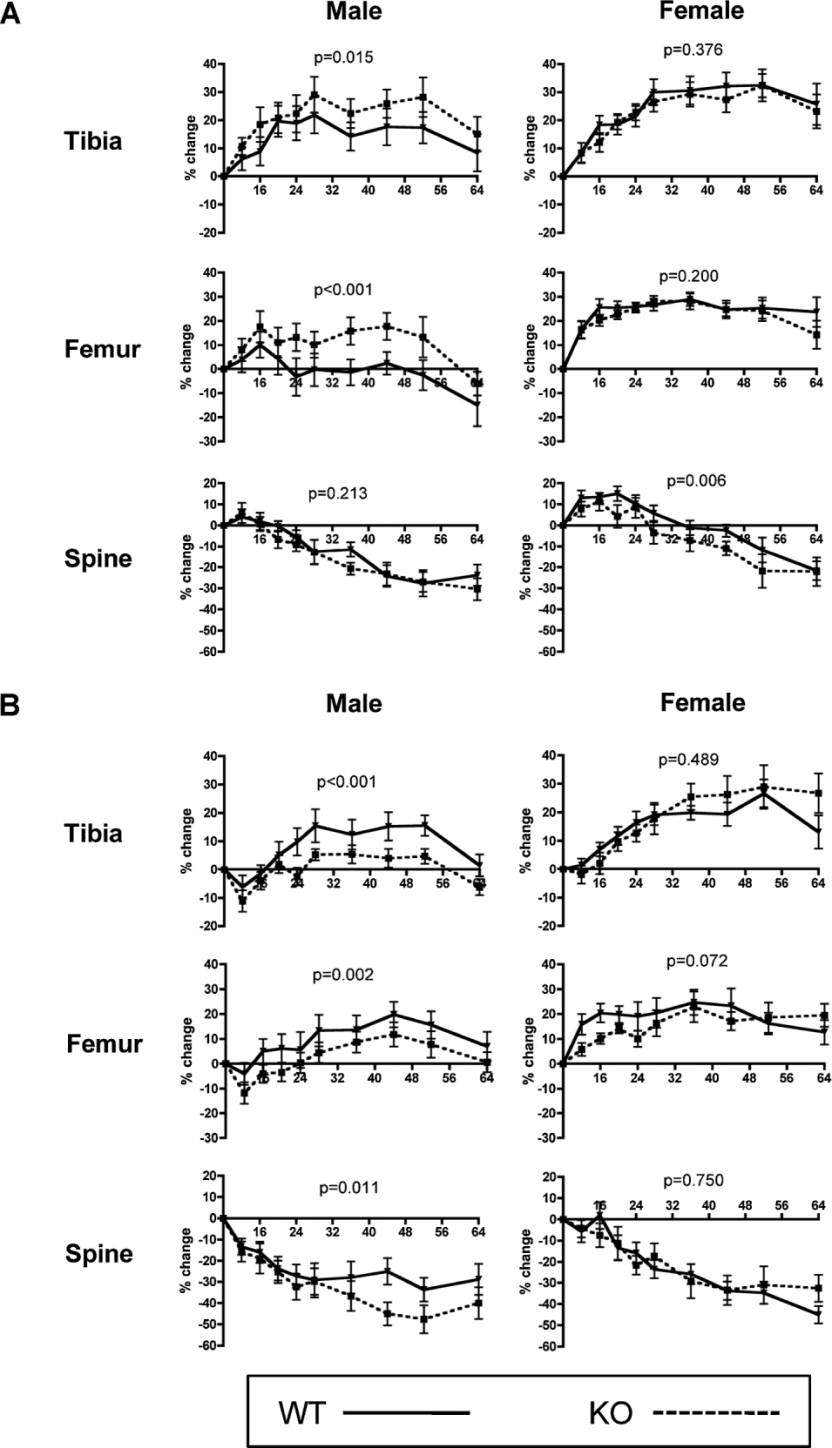
Osteoblast-targeted ETAR deletion alters BMD acquisition in aged male mice and is sex-steroid-specific. (*A*) Eugonadal and (*B*) hypogonadal WT (*solid line*) and KO (*dashed line*) male and female mice underwent longitudinal DXA analysis from 8 to 64 weeks of age. Percent change in BMD by DXA was determined in the proximal tibia, distal femur, and lumbar spine.

Significant differences in trabecular bone volume were first detected at 12 weeks of age based on µCT and histomorphometric data, although other trabecular parameters were significantly different at 8 weeks. The decline in BMD was examined next in older mice. The percent change in BMD was measured longitudinally in WT and KO male and female littermates by DXA between 8 and 64 weeks of age. In males, the KO group showed a significantly larger percent increase in tibial (*p* = 0.015) and femoral (*p* < 0.001) BMD acquisition than the WT group (Fig. 5*A*). No difference in spinal BMD accrual (*p* = 0.213) was observed between the groups. BMD accrual in tibia and femur was equivalent in females. Unlike the situation in males, a small but significant reduction in BMD accrual in KO versus WT mice was measured in the spine (*p* = 0.006).

The data showed gender-specific differences in bone responses to ETAR inactivation. Androgens might compensate for early losses in mice with ETAR-deleted osteoblasts, or they could even supplant the anabolic effects of ET-1 in bone. To test this hypothesis, castration was performed at 8 weeks of age, and bone accrual was examined between 8 and 64 weeks. Opposite to what was seen in the eugonadal experimental groups, castrated KO mice had reduced BMD accrual compared with WT mice in both the tibias (*p* < 0.001) and femurs (*p* = 0.002; Fig. 5*B*). Additionally, accelerated spine (*p* = 0.011) BMD loss occurred late in KO versus WT mice. Castration of female mice resulted in no significant differences between WT and KO animals at any sites. The data suggested interactions between ET-1 and androgen signaling in bone.

### Mechanisms of ET-1 and androgen in osteoblast activation

**Fig. 6:**
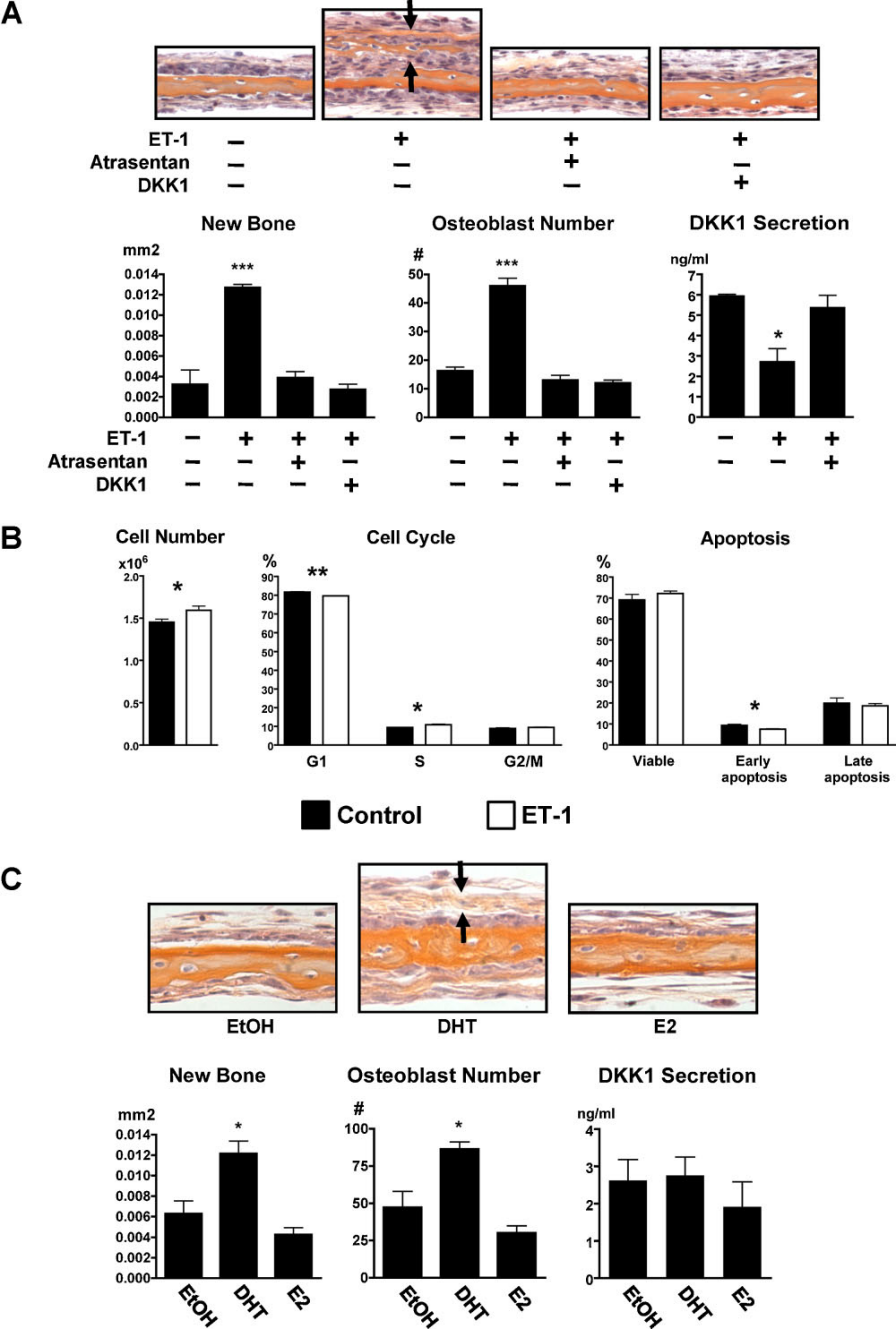
Mechanisms of ET-1 action in the osteoblast. (*A*) ET-1 (100 nM) increased bone formation (*orange staining between arrows*) and was blocked by either atrasentan (10 µm) or DKK1 (50 ng/mL) in the calvarial organ culture assay. ET-1 reduced DKK1 secretion from calvaria that was restored with atrasentan. (*B*) ET-1 increased cell number, proliferation, and survival of calvarial osteoblasts. (*C*) DHT (10 nM), but not E_2_ (10 nM), increased new bone formation (*orange staining between arrows*) and osteoblast number in the calvarial organ culture assay after 7 days of treatment. DKK1 production by calvaria was unchanged by sex steroids (*p ≤ 0.05).

ET-1 stimulation of osteoblasts and potential modulation by androgens were examined. In murine calvarial organ cultures, ET-1 significantly increased bone formation (0.0127 versus 0.0032 mm^2^, *p* < 0.001) and osteoblast number (46.0 versus 16.3 cells/0.57 mm of calvarial length, *p* < 0.001). This anabolic effect was blocked by either the ETAR antagonist atrasentan or DKK1 (Fig. 6*A*), consistent with previous reports.^4^, ^12^ ET-1 reduced DKK1 secretion by calvarial organ cultures (2.7 versus 5.9 ng/mL, *p* < 0.05; Fig. 6*A*), which results in activation of canonical Wnt signaling.^12^ ET-1 also increased cell density of primary calvarial osteoblast cultures after 48 hours of treatment (1.60 × 10^6^ versus 1.45 × 10^6^, *p* = 0.04; Fig. 6*B*). The increase in cell number could be due to increased proliferation, decreased apoptosis, or both. By flow cytometric cell cycle analysis, ET-1 modestly decreased the proportion of cells in the G_1_ resting phase (79.7% versus 81.7%, *p* = 0.002) and increased the proportion of cells in the S DNA synthesis phase (10.9% versus 9.4%, *p* = 0.012), indicating an increase in cell proliferation. ET-1 also had a modest pro-survival effect by decreasing the proportion of cells undergoing early apoptosis (7.5% versus 9.3%, *p* = 0.028).

The effects of sex steroids on calvarial organ cultures were determined next. Calvaria were treated with the nonaromatizable androgen 5α-dihydrotestosterone (DHT) or 17β-estradiol (E_2_) and compared with vehicle control. DHT, but not E_2_, significantly increased bone formation (0.0122 versus 0.0063 mm^2^, *p* < 0.05) and osteoblast numbers (86.3 versus 47.3 cells/0.57 mm of calvarial length, *p* < 0.05; Fig. 6*C*). DKK1 secreted from calvarial organ cultures did not change with DHT or E_2_, unlike the response to ET-1 (Fig. 6*C*). Flow cytometric analyses of primary calvarial osteoblasts showed no changes in cell proliferation or the proportion of cells undergoing apoptosis (data not shown).

**Fig. 7:**
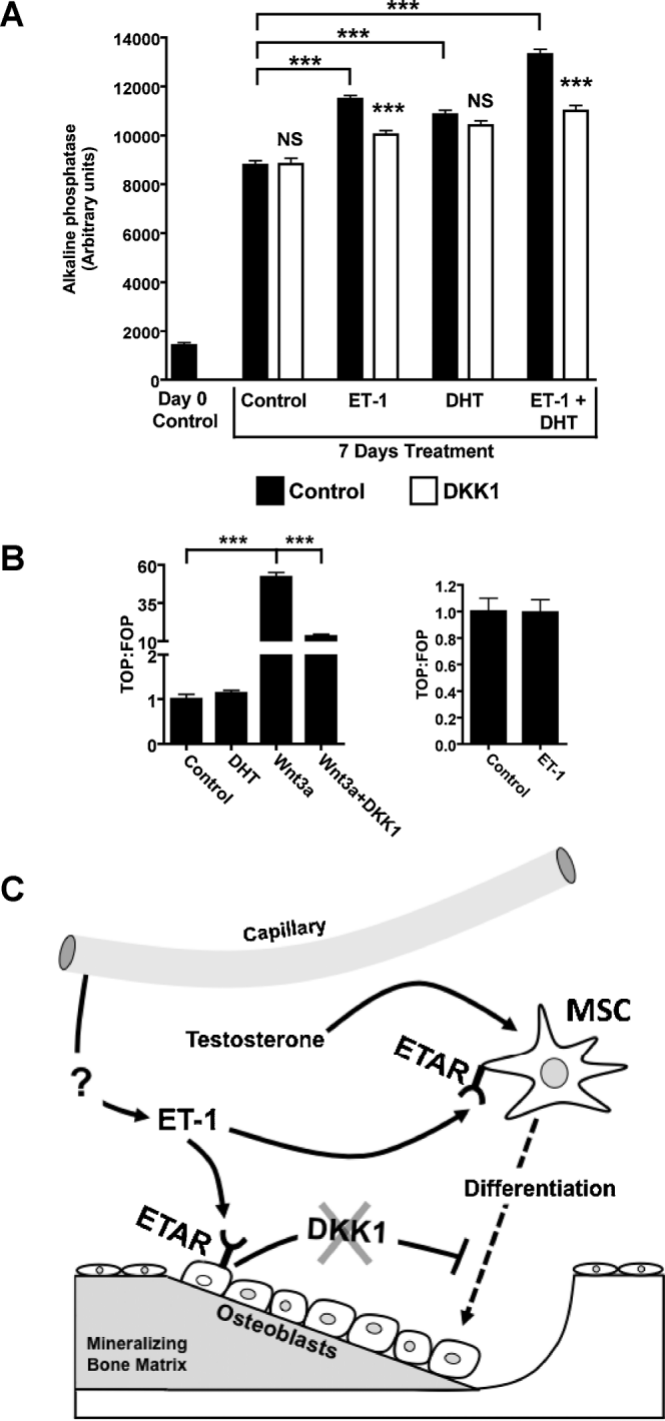
Mechanisms of ET-1 and DHT action in the osteoblast. (*A*) In MC3T3 osteoprogenitor cells, ET-1 (100 nM), DHT (10 nM), and combined treatment increased AP expression after 7 days of treatment. DKK1 (50 ng/mL) diminished AP staining in ET-1 and in combined ET-1 + DHT–treated cells. DKK1 did not change AP staining in DHT-treated cells. (*B*) Neither DHT nor ET-1 increased canonical Wnt signaling, as measured using Wnt reported vectors in isolated MC3T3 cells. Wnt3a (50 ng/mL) increased canonical Wnt signaling that was abrogated by DKK1 (50 ng/mL). (*C*) Model of ET-1, DHT, and DKK1 in regulating osteoblast activity. The source of ET-1 in the bone microenvironment is most likely vascular endothelial cells of adjacent capillaries. ET-1 promotes osteoprogenitor differentiation through direct mechanisms independent of Wnt activation and indirectly by lowering the tonic inhibition of DKK1 secreted in mature osteoblasts. Testosterone complements the actions of ET-1 by increasing osteoprogenitor differentiation through unclear mechanisms (*p ≤ 0.05; ***p ≤ 0.001).

Although both ET-1 and DHT increased bone formation significantly, the modest increase in osteoblast cell number by ET-1 and no increase with DHT suggested that differentiation of osteoprogenitor cells to matrix-forming osteoblasts is responsible for enhanced bone formation with ET-1 and DHT. To test this, the MC3T3 preosteoblastic cell line was treated with ET-1 and DHT for 7 days and tested for expression of alkaline phosphatase (AP), a marker of osteoblastic differentiation (Fig. 7*A*). ET-1 and DHT significantly increased AP expression. The combination of ET-1 plus DHT increased AP expression more than ET-1 alone. The contribution of canonical Wnt signaling in ET-1- and DHT-mediated osteoblastic differentiation was assessed using DKK1 in combination treatments. DKK1 reduced ET-1 and ET-1 + DHT–mediated differentiation. However, DKK1 did not change AP expression in DHT-treated preosteoblasts.

The direct, DKK1 independent effects of ET-1 and DHT on Wnt signaling were determined in preosteoblasts, a stage in which Wnt signaling is active and critical for differentiation. Differentiating MC3T3 cells were treated with ET-1 or DHT, and Wnt signaling was assessed with reporter constructs. Neither ET-1 nor DHT activated canonical Wnt signaling in these osteoprogenitors (Fig. 7*B*). Therefore, suppression by ET-1 of DKK1 secretion from mature osteoblasts thus was essential for control of Wnt signaling in osteoprogenitors.

Expression analyses were performed to further clarify when and where ET-1 and DKK1 are expressed in bone. DKK1 is present in the conditioned medium of calvarial organ cultures, which contains osteoblasts at all stages of differentiation from osteoprogenitors to osteocytes. However, DKK1 was not detected in the conditioned medium of dividing primary osteoblasts or MC3T3 cells in an ELISA with a detection limit of 34 pg/mL (data not shown). The data suggest that DKK1 is a marker of late osteoblasts. ET-1 was not detected in the conditioned media from a variety of osteoblast-containing cultures (data not shown).

## Discussion

The endothelin A receptor involved in the pathologic bone formation of osteoblastic bone metastasis has now been shown to regulate normal bone remodeling. ETAR does not regulate all aspects of bone homeostasis and is restricted to the postnatal period and to trabecular bone of the appendicular skeleton. Neither spine trabecular bone volume nor cortical bone was substantially altered by ETAR osteoblast inactivation in osteoblasts. The structural basis for reduced trabecular bone volume in ETAR KO animals appeared to differ between males and females. In males, trabecular thickness and trabecular number both contributed to reduced trabecular bone volume with ETAR inactivation. Trabecular thickness was either unchanged or paradoxically higher in female KO mice, indicating that the degree of reduced trabecular numbers in female KO mice negated any increases in trabecular thickness. The restricted phenotype of reduced trabecular bone volume in appendicular sites is supported by quantitative trait loci (QTL) analyses of humans and mice, demonstrating that distinct genetic loci regulate bone mass in site-specific and gender-specific manners.^28^–^30^ In fact, ETAR is located in the 40-cM region of mouse chromosome 8 near a QTL frequently identified as regulating bone mass.^29^, ^31^–^33^

The actions of ET-1 on the osteoblast depended on the stage of differentiation. ET-1 increased differentiation of MC3T3 preosteoblasts and proliferation and survival of dividing calvarial osteoblasts. These effects were independent of canonical Wnt signaling and DKK1. DKK1 was not detected in these two cell types, supporting it as a marker of mature osteoblasts, consistent with another report.^34^ DKK1 was secreted abundantly in calvarial organ cultures, which contain the full complement of bone-forming cells from osteoprogenitors to osteocytes and bone matrix. In this experimental setting, ET-1 decreased DKK1. ET-1 therefore has dual functions in bone: (1) It increases the pool of osteoprogenitor cells, and (2) it increases Wnt signaling in early osteoblasts by decreasing secretion of paracrine DKK1 from mature osteoblasts.

Another potential function of the endothelin axis is to cooperate with androgens in bone. Changes in bone density were examined beginning at sexual maturity in castrated and eugonadal mice. In eugonadal male mice, the rate of bone acquisition in appendicular BMD was higher in KO mice than in WT controls but reversed in hypogonadal male mice. Since the absolute BMD in young KO mice was lower, these data suggested that androgens might compensate for earlier losses in appendicular skeletal BMD owing to ETAR inactivation or might even replace the actions of ET-1. Another interpretation is that ET-1 desensitizes bone to the effects of testosterone. Either way, it is clear that the differentiating effects of ET-1 and DHT occur through different mechanisms because DKK1 blocked the differentiating effects of ET-1 but not DHT. Other cooperative mechanisms between androgen signaling and Wnt signaling may occur. The androgen receptor (AR), but not estrogen receptor α or other nuclear receptors, physically interacts with β-catenin in an androgen-dependent manner.^35^, ^36^ Activation of either AR or Wnt signaling mutually increases the signaling activity of the other signaling pathway. DHT increased β-catenin nuclear localization and Wnt signaling in some cells.^36^, ^37^ Conversely, β-catenin increased DHT-mediated transcription of AR targets.^35^

Our results address the significance of the endothelin axis in normal bone development. A number of different cell types secrete ET-1, but the data presented here demonstrated that the osteoblast is not the source in bone. Vascular endothelial cells are among the most abundant producers of ET-1, where it functions in maintaining vascular tone.^1^ During endochondral bone formation, osteoblast precursors invade the matrix scaffold laid down by chondrocytes within the primary spongiosa, an area rich in vascular endothelium. ET-1 from these cells could activate ETAR on adjacent osteoprogenitor cells. However, since the bone phenotype from ETAR inactivation was first detected in 12-week-old adults during a time when intense osteoblast activity is not focused around the growth plate, other areas of bone remodeling of adulthood are likely where osteoblasts “couple” to the vasculature. The bone remodeling compartment (BRC) was first reported in 2001 and is described as a sinus involved in trabecular bone remodeling.^38^ Groups of matrix-producing osteoblasts are covered by a thin canopy of lining cells that are intimately associated with capillary vascular endothelial cells. The osteoblastic response to the vasculature would encourage new bone formation in areas of adequate oxygen and nutrient supply. The idea of coupling osteoblasts with vasculature is not novel. Others have proposed that hypoxia stimulates osteoblast vascular endothelial growth factor secretion, resulting in enhanced angiogenesis and bone repair.^39^, ^40^

The data presented here have important implications for men who undergo androgen-deprivation therapy for advanced prostate cancer. Chemical or surgical castration to reduce circulating androgens is a key component of prostate cancer therapy. Androgen deprivation delays recurrence of disease but significantly increases the risk of clinically significant spine, hip, and forearm fractures.^41^–^43^ Although ETAR antagonists hold promise to reduce the progression of prostate cancer bone metastases, additional morbidity and cost may result owing to an increased fracture risk. Detailed bone analyses have not yet been performed during clinical trials of endothelin receptor antagonists. Additional implications of these results are significant because most men who receive androgen-deprivation therapy are neither evaluated nor treated for bone loss.^44^, ^45^ The ETAR antagonists bosentan and ambrisentan are currently approved for treatment of advanced pulmonary hypertension,^46^, ^47^ but the impact of these medications in this population is unknown.

In summary, the ET-1/ETAR axis is an important regulator of osteoblast activity and trabecular bone remodeling (Fig. 7*C*). ET-1 increases osteoprogenitor proliferation and differentiation to mature bone matrix–secreting osteoblasts. ET-1-targeted downregulation of DKK1 secreted during late osteoblast development is key in reducing canonical Wnt signaling inhibition of osteoprogenitor cells. DHT also promotes in vitro osteoprogenitor cell differentiation through a mechanism that is different from ET-1. The cooperation of ET-1 and androgen in vivo is clearly more complex. The contribution of vascular endothelial-secreted ET-1 and osteoclasts in mediating the cooperative effects of ET-1 and androgen is an important area of future study.
